# Buckling Analysis of Functionally Graded GPL-Reinforced Composite Plates Under Combined Thermal and Mechanical Loads

**DOI:** 10.3390/ma18030567

**Published:** 2025-01-26

**Authors:** Jin-Rae Cho

**Affiliations:** Department of Naval Architecture and Ocean Engineering, Hongik University, Sejong 30016, Republic of Korea; jrcho@hongik.ac.kr

**Keywords:** GPL-reinforced composite, coupled buckling problem, critical buckling temperature rise, critical buckling load, parametric investigation

## Abstract

The buckling-like mechanical behavior of functionally graded graphene platelet-reinforced composite (FG-GPLRC) structures is increasingly attracting research attention. However, buckling behavior has previously been studied separately as thermal buckling and mechanical buckling. In this context, this study investigates the buckling behavior of FG-GPLRC plates under combined thermal and mechanical loads. The coupled buckling problem is formulated according to the minimum potential energy theorem using first-order shear deformation theory (FSDT). In addition, the problem is approximated by the 2-D natural element method (NEM), and the resulting coupled eigen matrix equations are derived to compute the critical buckling temperature rise (CBTR) and the mechanical buckling load. The developed numerical method can solve thermal, mechanical, and coupled thermo-mechanical buckling problems, and its reliability is examined through convergence and benchmark tests. Using the developed numerical method, the buckling behavior of FG-GPLRC plates under thermal and mechanical buckling loads is examined in depth with respect to the key parameters. In addition, a comparison with functionally graded CNT-reinforced composite (FG-CNTRC) plates is also presented.

## 1. Introduction

Laminated composite structures have been widely adopted in various engineering applications due to their excellent mechanical properties, e.g., a high weight–stiffness ratio [1]. Despite their many advantages, traditional laminated composite structures suffer from several severe problems, such as delamination and micro-cracking [2]. These critical problems are known to be triggered by the discontinuity in the material properties across the layer interfaces [3]. To overcome these critical problems, the concept of functionally graded material (FGM) was introduced in the late 1990s, where material properties vary continuously with the thickness [4]. The material continuity in FGM is enforced by inserting a graded layer, in which a material property changes continuously from that of one layer to that of the other layer, between two distinct layers [5]. Besides remedying critical problems, FGM can further enhance the target mechanical behavior by appropriately designing the material composition distribution across the thickness in the graded layer [6]. The composite structures fabricated according to the FGM concept are called functionally graded composite structures, and they are extensively used in mechanical, aeronautical, bio-technology, sports, and many other engineering fields [7].

Meanwhile, composite structures have been advanced dramatically with the advent of carbon nanofillers, graphene platelets (GPLs), and carbon nanotubes (CNTs), which are called twenty-first century materials and have been spotlighted next-generation materials [8,9]. Carbon nanofillers have been spotlighted as a next-generation advanced filler due to their extraordinary mechanical properties. It has been reported that the elastic modulus of graphene platelets is about 1000 times stronger than that of steel, indicating that the material properties of conventional composite structures could be dramatically improved with only a tiny amount of carbon nanofillers [9]. Nevertheless, the addition of these nanofillers to composite structures is limited to small amounts owing to their currently prohibitive cost. To mitigate this issue, the concept of FGM has been applied to carbon-reinforced nanocomposite structures, and several purposeful primitive functionally graded dispersion patterns of carbon nanofillers across the thickness were presented in [10]. As the total amount of reinforced carbon nanofillers can be reduced by appropriately dispersing these nanofillers, functionally graded graphene-reinforced composite structures can be produced via the FGM concept.

Graphene-reinforced composites are generally fabricated in the form of beam, plate, and shell structures, so the success of their practical applications strongly relies on the accurate understanding of their mechanical behaviors. In this regard, their mechanical behaviors are related to the material and geometry parameters and the loading and constraint conditions [11]. Studies of these behaviors have been conducted analytically, mainly using traditional plate and shell theories and using finite element methods and other recent numerical methods for numerical solutions. Buckling during these mechanical responses greatly reduces the structure’s load-carrying capacity before it reaches its ultimate strength [12]. In this respect, the buckling behavior of graphene-reinforced nanocomposite structures is considered an important research subject. Buckling is generally caused by compressive in-plane loads, which are induced by mechanical or/and thermal loads.

Regarding the buckling of graphene-reinforced composite structures, Zhang et al. [13] examined the buckling response of graphene sheets embedded in elastic foundations by applying the kp-Ritz method to the classical plate theory incorporated with the non-local elasticity theory. Yang et al. [14] examined the buckling behavior of a graphene sheet/substrate system both via molecular mechanics simulations and theoretical analysis. Shen et al. [15] applied a two-stage perturbation technique to the higher-order shear deformation plate theory to analyze the thermal post-buckling of GPLRC-laminated plates placed on an elastic foundation. Wu et al. [16] parametrically investigated the thermal buckling and post-buckling of functionally graded multilayer composite plates by introducing a differential quadrature (DQ) iteration technique to the FSDT. Barati and Zenkour [17] analyzed the post-buckling behavior of geometrically imperfect GPLRC beams placed on nonlinear hardening foundation by applying Galerkin’s method. Mirzaei and Kiani [18] parametrically investigated the thermal buckling behaviors of GPLRC-laminated plates using a non-uniform rational B-spline (NURBS)-based isogeometric finite element method (FEM). Wang et al. [19] parametrically examined the torsional buckling of FG-GPLRC cylindrical shells by FEM with respect to the cutout parameters. Yang et al. [20] investigated the buckling behavior of FG-GPLRC plates with porosities by applying the Chebyshev–Ritz method to the FSDT with respect to both the porosity and the GPL dispersion types. Mao and Zhang [21] analyzed the buckling and post-buckling of multilayer FG-GPLRC piezoelectric plates by applying the DQ method and a direct iterative technique to the FSDT. Kiani [22] presented the buckling analysis of FG-GPLRC-laminated conical shells by applying the general DQ method to the FSDT incorporated with von Kármán nonlinearity. Moayedi et al. [23] presented a thermal buckling analysis of a GPLRC doubly curved cylindrical panel using a numerical-based 2-D general quadrature method incorporated with a nonlocal strain gradient theory. Safaei et al. [24] presented the thermal buckling of carbon-form sandwich beams with composite faces under axial compression. Wang and Zhang [25] examined the thermal buckling and post-buckling behaviors of GPLRC porous nanocomposite beams by considering the temperature dependence of the material properties. Cho [26] investigated the buckling behavior of FG-GPLRC cylindrical panels with internal cracks with respect to key parameters related to the cracks, GPL, and cylindrical panels by applying a 2-D natural element method (NEM) to the FSDT. Regarding non-GPLRC structures, Yu et al. [12] and Abolghasemi et al. [27] studied the coupled thermo-mechanical buckling of metal–ceramic functionally graded plates.

According to the above literature survey, previous studies on the buckling of FG-GPLRC structures have been divided into mechanical buckling and thermal buckling, and most of them were conducted separately, except for a few studies on the coupling buckling of non-GPLRC structures. In other words, buckling due to a combination of mechanical and buckling loads has rarely been investigated in FG-GPLRC structures, even though it is mostly caused by a combined load rather than a single load [28]. The buckling responses of FG-GPLRC structures under coupled loads must, therefore, be investigated to achieve the successful practical application of these structures. In this context, the goal of this study was to investigate the buckling behavior of FG-GPLRC plates in an epoxy matrix that combines mechanical and thermal loads by developing effective numerical analysis methods. The buckling problem under coupled thermo-mechanical loading was formulated according to the FSDT [29], and the effective material properties of FG-GPLRC plates were calculated by volume fraction-based homogenization methods [30]. The displacement field in the FSDT was approximated by the 2-D NEM. This mesh-free method makes grid generation and adaptation easier and improves the accuracy compared to traditional FEMs [31]. The resulting eigenvalue matrix equations were solved by Lanczos transformation and the Jacobi iteration method. The convergence and accuracy of the developed numerical method were examined through benchmark tests, and the uni- and bi-axial buckling responses of the FG-GPLRC plates in the epoxy matrix were investigated in depth with respect to the associated key parameters.

## 2. Functionally Graded GPL-Reinforced Plate

**Figure 1:**
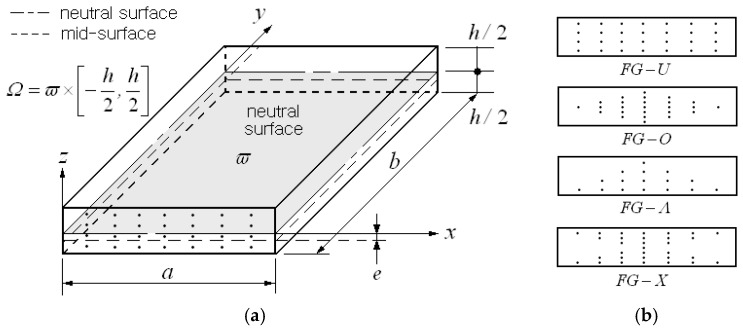
A GPL-reinforced plate: (**a**) parameters and dimensions; (**b**) four GPL distribution patterns.

Figure 1a represents a rectangular nanocomposite plate in which graphene platelets (GPLs) are mixed into an epoxy matrix according to a specific thickness-wise distribution pattern. The geometry of the plate is characterized by its mid-surface ω having two sides, a and b, and a uniform thickness h; thus, the material area can be represented by $\Omega =\omega \times \left[-h/2,h/2\right]$. The thickness-wise GPL distribution may be random or purposeful, as illustrated in Figure 1b, and these primitive patterns are called functionally graded (FG) distribution patterns [32]. These FG distribution patterns were not generated during the fabrication process and only proposed to investigate the changes in the mechanical behavior of these patterns. These distribution patterns can, of course, be fabricated by the current manufacturing technology. Graphene platelets are uniform in the FG-U throughout its thickness, concentrated at the mid-surface in the FG-O, concentrated at the bottom in the FG-Λ, and concentrated at the top and bottom in the FG-X. If the material configuration is not symmetrical to the mid-surface, the neutral surface $\bar{\varpi }$ deviates from the mid-surface by a vertical distance e.

The material composition of dual-phase composites is usually expressed by the volume fractions of the constituent particles. Letting $V_{m}\left(z\right)$ and $V_{GPL}\left(z\right)$ be the volume fractions of the matrix and GPL, which are functions of co-ordinate z, both satisfy the physical constraint given by

(1) $$V_{GPL}\left(z\right)+V_{m}\left(z\right)=1,\;-h/2\leq z\leq h/2$$

Thanks to this relationship, either one is enough to identify two volume fractions in the thickness direction. This study utilized the GPL volume fraction $V_{GPL}\left(z\right)$, which is mathematically expressed as

(2) $$V_{GPL}\left(z\right)=\left\{\begin{array}{ll} V_{GPL}^{*}, & \mathrm{FG}\;-\;U\;\\ \left(2-4\left|z\right|/h\right)V_{GPL}^{*}, & \mathrm{FG}-O\;\\ \left(1-2z/h\right)V_{GPL}^{*}, & \mathrm{FG}-\Lambda \\ \left(4\left|z\right|/h\right)V_{GPL}^{*}, & \mathrm{FG}-X \end{array}\right.$$

for these four functionally graded GPL dispersion types. Here, $V_{GPL}^{*}$ is the total GPL volume fraction determined by $V_{GPL}^{*}=g_{GPL}^{*}/\left[g_{GPL}^{*}+\rho_{GPL}\left(1-g_{GPL}^{*}\right)/\rho_{m}\right]$ using the total mass fraction $g_{GPL}^{*}$ and densities $\rho_{GPL}$ and $\rho_{m}$ of the GPL and matrix, respectively.

The mechanical responses of dual-phase nanocomposites in the macroscopic sense are usually evaluated by the effective material properties. Graphene platelets are usually assumed to be uniformly dispersed in the matrix and modeled as a rectangular solid with length $l_{GPL}$, width $w_{GPL}$, and thickness $t_{GPL}$. Thus, GPLRC plates are considered isotropic, and the effective Poisson’s ratio ν and thermal expansion coefficient $\alpha_{t}$ can be determined using

(3) $$\nu =V_{GPL}\nu_{GPL}+V_{m}\nu_{m}$$

(4) $$\alpha_{T}=V_{GPL}\alpha_{t,GPL}+V_{m}\alpha_{t,m}$$

based on the simplest linear rule of the mixtures [33]. Meanwhile, the effective elastic modulus E is calculated as

(5) $$E=\frac{3}{8}\cdot \frac{1+\xi_{L}\eta_{L}V_{GPL}}{1-\eta_{L}V_{GPL}}E_{m}+\frac{5}{8}\cdot \frac{1+\xi_{T}\eta_{T}V_{GPL}}{1-\eta_{T}V_{GPL}}E_{m}$$

using the Halpin–Tsai micromechanics approach [14,30]. Here, the two parameters $\eta_{L}$ and $\eta_{T}$ are defined by

(6) $$\eta_{L}=\frac{E_{GPL}-E_{m}}{E_{GPL}+\xi_{L}E_{m}},\;\eta_{T}=\frac{E_{GPL}-E_{m}}{E_{GPL}+\xi_{T}E_{m}}$$

using the elastic moduli $E_{GPL}$ and $E_{m}$ of the GPL and matrix, respectively, and the dimensional ratios $\xi_{L}=2l_{GPL}/t_{GPL}$ and $\xi_{T}=2w_{GPL}/t_{GPL}$. Then, the effective material properties corresponding to the above FG distribution patterns can be theoretically determined, although the determined material properties were not validated by experiments.

The effective material properties described above are a function of co-ordinate z, and those become non-symmetrical when the GPL volume fraction $V_{GPL}\left(z\right)$ is not symmetric. In this case, the vertical location e of the neutral surface from the mid-surface is calculated as follows

(7) $$e=\int_{-h/2}^{h/2}zE\left(z\right)dz/\int_{-h/2}^{h/2}E\left(z\right)dz$$

## 3. First-Order Shear Deformation Plate Theory

The displacement field $u={\left\{u,v,w\right\}}^{T}$ of the FG-GPLRC plate subjected to coupled thermal and mechanical loads is expressed as

(8) $${\left\{\begin{array}{c} u\\ v\\ w \end{array}\right\}}_{\left(x,y,z\right)}={\left\{\begin{array}{c} u_{0}\\ v_{0}\\ w_{0} \end{array}\right\}}_{\left(x,y\right)}+z\cdot {\left\{\begin{array}{c} \theta_{x}\\ \theta_{y}\\ 0 \end{array}\right\}}_{\left(x,y\right)}$$

using the FSDT. As a concise description, nodal in-plane displacement vector $a={\left\{u_{0},v_{0},w_{0}\right\}}^{T}$ and nodal rotational displacement vector $r={\left\{\theta_{x},\theta_{y}\right\}}^{T}$ are combined as a nodal displacement vector $b={\left(u_{0},v_{0},w_{0},\theta_{x},\theta_{y}\right)}^{T}$. The strain–displacement relationship is then expressed as

(9) $$\varepsilon =\left\{\begin{array}{c} \varepsilon_{xx}\\ \varepsilon_{yy}\\ 2\varepsilon_{xy} \end{array}\right\}=\left\{\begin{array}{c} u_{0,x}\\ v_{0,y}\\ u_{0,y}+v_{0,x} \end{array}\right\}+z\cdot \left\{\begin{array}{c} \theta_{x,x}\\ \theta_{y,y}\\ \theta_{x,y}+\theta_{y,x} \end{array}\right\}=A_{L}b$$

(10) $$\gamma =\left\{\begin{array}{c} \gamma_{yz}\\ \gamma_{zx} \end{array}\right\}=\left\{\begin{array}{c} \theta_{y}+w_{0,y}\\ \theta_{x}+w_{0,x} \end{array}\right\}=A_{s}b$$

with an in-plane strain vector ε and out-of-plane transverse strain vector γ, where $A_{L}$ and $A_{s}$ indicate the $\left(3\times 5\right)$ and $\left(2\times 5\right)$ partial derivative matrices defined by

(11) $$A_{L}=\left[\begin{array}{ccccc} A_{x} & 0 & 0 & z\cdot A_{x} & 0\\ 0 & A_{y} & 0 & 0 & z\cdot A_{y}\\ A_{y} & A_{x} & 0 & z\cdot A_{y} & z\cdot A_{x} \end{array}\right]$$

(12) $$A_{s}=\left[\begin{array}{ccccc} 0 & 0 & A_{y} & 0 & 1\\ 0 & 0 & A_{x} & 1 & 0 \end{array}\right]$$

with $A_{x}$ being ∂/∂x and $A_{y}$ being ∂/∂y. The stress–strain constitutive relations, taking into account the thermal contribution, are then expressed as

(13) $$\sigma =\frac{E}{1-\nu^{2}}\left[\begin{array}{ccc} 1 & \nu & 0\\ \nu & 1 & 0\\ 0 & 0 & \left(1-\nu \right)/2 \end{array}\right]\left\{\begin{array}{c} \varepsilon_{xx}-\alpha_{t}\Delta T\\ \varepsilon_{yy}-\alpha_{t}\Delta T\\ 2\varepsilon_{xy} \end{array}\right\}=D\left[A_{L}b-\alpha_{t}\Delta T1\right]$$

(14) $$\tau =\kappa \left[\begin{array}{cc} G & 0\\ 0 & G \end{array}\right]\left\{\begin{array}{c} \gamma_{yz}\\ \gamma_{zx} \end{array}\right\}=D_{s}H_{s}b$$

with $\sigma ={\left\{\sigma_{xx},\sigma_{yy},\sigma_{xy}\right\}}^{T}$, $\tau ={\left\{\tau_{yz},\tau_{zx}\right\}}^{T}$ and, $1={\left\{1,1,0\right\}}^{T}$ and the shear correction factor $\kappa =5/\left(6-\nu \right)$.

**Figure 2:**
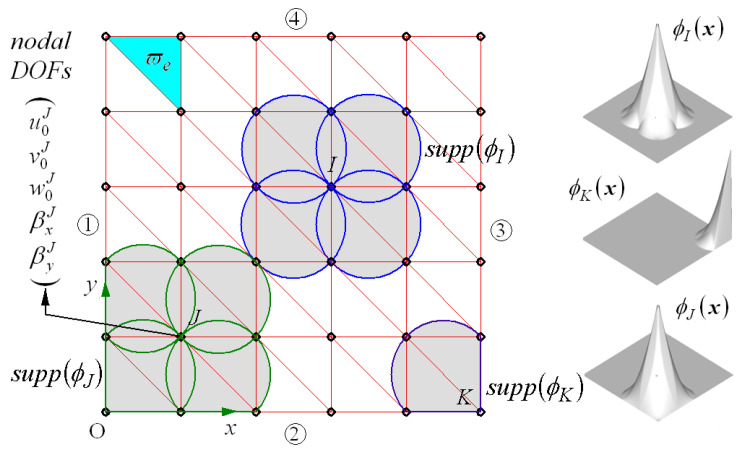
A uniform NEM and Laplace interpolation (L/I) functions.

Referring to Figure 2, the neutral surface $\bar{\varpi }$ of the plate is uniformly divided into three-node Delaunay triangles to approximate the displacement field. The approximated displacement field $u^{h}\left(x,y,z\right)$ in the natural element method (NEM) is then expressed as

(15) $$u^{h}\left(x\right)=\sum_{J=1}^{N}\left(a_{J}+z\;r_{J}\right)\;\varphi_{J}\left(x,y\right)$$

using L/I functions $\varphi_{J}\left(x,y\right)$ [31]. Here, $b_{J}=\left(a_{J},\;r_{J}\right)$ indicates the nodal displacement vector at nodes J in the NEM grid shown in Figure 2, which consists of M Delaunay triangles and N nodes, where supp($\varphi_{J}$) (i.e., the grayed region) denotes the domain of the *J*-th L/I function $\varphi_{J}$ in which $\varphi_{J}$ varies from unity at node *J* to zero around the edge.

 Substituting Equation (15) into Equations (9) and (10) leads to the approximated in-plane strain $\varepsilon^{h}$ and transverse shear (T/S) strain $\gamma^{h}$ given by

(16) $$\varepsilon^{h}=\sum_{J=1}^{N}A_{L}\varphi_{J}b_{J}=\sum_{J=1}^{N}B_{L}\;b_{J}$$

(17) $$\gamma^{h}=\sum_{J=1}^{N}A_{s}\varphi_{J}b_{J}=\sum_{J=1}^{N}B_{s}^{J}b_{J}$$

where $B_{L}^{J}=A_{L}\varphi_{J}$ and $B_{s}^{J}=A_{s}\varphi_{J}$.

As represented in Figure 2, L/I functions are non-vanishing only over their supports supp($\varphi_{J}$) indicated by graded regions; thus, the approximated displacement field $u^{h}\left(x\right)$ is continuous support-wise. Therefore, it belongs to the $C^{0}$—continuous function, and the T/S strain in Equation (17) may encounter shear locking when the thickness of the bending-dominated plate is small [34,35]. This phenomenon can be effectively suppressed by the indirect interpolation of T/S strain according to the MITC(mixed interpolation of tensorial components)3+ shell approach [36]. Refer to [37] for details on the numerical implementation of this approach to the NEM. Letting ${\hat{B}}_{e}$ and $b_{e}=\left\{b_{1}^{e},b_{2}^{e},b_{3}^{e}\right\}$ be a $\left(2\times 15\right)$ triangular-wise partial derivative matrix and a triangle-wise nodal displacement vector, respectively, the interpolated locking-free T/S stain is expressed as

(18) $$\gamma_{e}^{h}={\hat{B}}_{e}b_{e}$$

Next, by substituting Equations (16)–(18) into Equations (13) and (14), one can compute the in-plane stress $\sigma^{h}$ and the T/S stress $\tau^{h}$ given by

(19) $$\sigma^{h}=\sum_{J=1}^{N}DB_{L}^{J}\;b_{J}-D\alpha_{t}\Delta T1$$

(20) $$\tau^{h}=\sum_{e=1}^{M}D_{s}{\hat{B}}_{e}b_{e}$$

## 4. Analysis of Coupled Thermo-Mechanical Buckling

**Figure 3:**
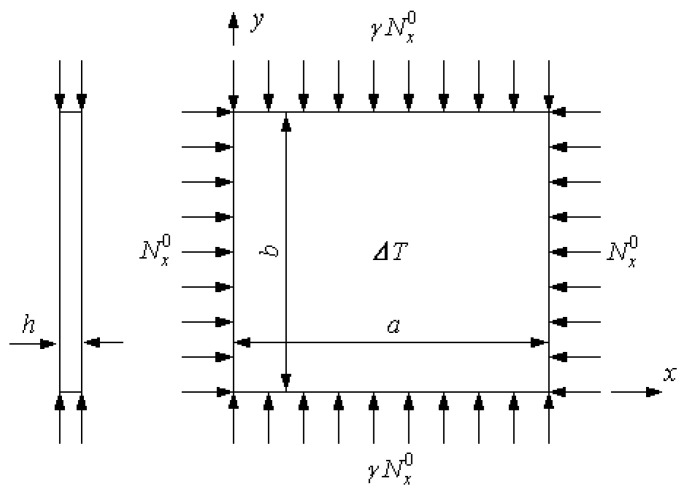
An FG-GPLRC plate under in-plane loading and uniform temperature rise.

As shown in Figure 3, an FG-GPLRC plate was subjected to the normal forces $N_{x}^{0}$ and $N_{y}^{0}$ along the plate edge and a uniform temperature rise ΔT. The strain energy of the plate is denoted by U and the work performed by applied edge forces and internal forces are denoted by $W_{\Gamma }$ and $W_{I}$, respectively. Then, the total potential energy Π is defined as

(21) $$\Pi =U-W_{\Gamma }-W_{2}$$

where the strain energy δU of the FG-GPLRC plate is computed as

(22) $$\delta \;U=\int_{-h/2+e}^{h/2-e}\int_{\bar{\varpi }}\delta \varepsilon^{T}\sigma \;dAdz$$

with the vertical distance e between the middle and neutral surfaces. Meanwhile, the work performed $W_{\Gamma }$ by the external edge loads is computed as

(23) $$W_{\Gamma }=\int_{\partial \bar{\varpi }}\left(N_{x}^{0}u_{0}+N_{y}^{0}v_{0}\right)d\Gamma$$

where $u_{0}$ and $v_{0}$ are the displacement components at the edges of the plate. The work performed $W_{I}$ by the internal forces is defined by

(24) $$W_{I}=\frac{1}{2}\int_{h/2+e}^{h/2-e}\int_{\bar{\varpi }}{\left\{\begin{array}{c} w_{,x}\\ w_{,y} \end{array}\right\}}^{T}\left[\begin{array}{cc} N_{x} & N_{xy}\\ N_{xy} & N_{y} \end{array}\right]\left\{\begin{array}{c} w_{,x}\\ w_{,y} \end{array}\right\}dAdz$$

where the resulting in-plane forces $N_{\alpha \beta }\left(\alpha ,\beta =x,y\right)$ are defined by

(25) $$N_{\alpha \beta }=\int_{-h/2\_e}^{h/2-e}\sigma_{\alpha \beta }dz$$

By substituting Equations (16)–(20) into Equation (31) using Equations (22)–(25), one can obtain the matrix equation given by

(26) $$\left(K_{\sigma }+\sum_{e=1}^{M}K_{s}^{e}-K_{g}\right)\;\bar{d}=\Delta TF_{th}+\lambda F_{me}$$

according to the minimum potential energy theorem [27]. Here, the stiffness matrices $K_{\sigma }$ and $K_{s}^{e}$, the geometric stiffness matrix $K_{g}$, and two load vectors $F_{th}$ and $F_{me}$ are defined by

(27) $$K_{\sigma }=\int_{-h/2+e}^{h/2-e}\int_{\bar{\varpi }}B^{T}DB\;dAdz$$

(28) $$K_{s}^{e}=\int_{-h/2+e}^{h/2-e}\int_{{\bar{\varpi }}_{e}}B_{e}^{T}{\hat{D}}_{s}B_{e}\;dAdz$$

(29) $$K_{g}=\int_{-h/2+e}^{h/2-e}\int_{\bar{\varpi }}G^{T}\left[\begin{array}{cc} N_{x} & N_{xy}\\ N_{xy} & N_{y} \end{array}\right]G\;dAdz$$

(30) $$F_{th}=\int_{-h/2+e}^{h/2-e}\int_{\bar{\varpi }}B^{T}D\left(\alpha_{t}\Delta T1\right)dAdz$$

(31) $$F_{me}=\int_{\partial \bar{\varpi }}\left(N_{x}^{0}u^{0}+N_{y}^{0}v^{0}\right)d\Gamma$$

with the $\left(2\times N\right)$ matrix G defined by

(32) $$G=\left[\left(\begin{array}{c} \varphi_{1,x}\\ \varphi_{1.y} \end{array}\right),\left(\begin{array}{c} \varphi_{2,y}\\ \varphi_{2,y} \end{array}\right),\ldots ,\left(\begin{array}{c} \varphi_{N,x}\\ \varphi_{N,y} \end{array}\right)\right]$$

In a numerical analysis of coupled thermo-mechanical buckling, the plate is subjected to an initial thermal or mechanical loading. First, assume that the plate is subjected to in-plate mechanical loading at its edges, and the thermal buckling temperature is to be computed. By excluding the geometric stiffness matrix $K_{g}$ from Equation (26), one can solve the pre-buckling displacement $\bar{d}$ due to both mechanical and thermal loads using [12]

(33) $$\left(K_{\sigma }+K_{s}\right)\;\bar{d}=\Delta TF_{th}+\lambda F_{me}$$

where $K_{s}=\sum_{e=1}^{M}K_{s}^{e}$. The critical buckling temperature rise (CBTR) $\Delta T_{cr}$ can then be computed using the following eigenvalue matrix:

(34) $$\left(K_{\sigma }+K_{s}-K_{g}\right)\;\bar{d}=0$$

Here, the geometric stiffness matrix $K_{g}$ is related to the thermal and mechanical pre-buckling displacements. Equation (34) can, therefore, be rewritten as

(35) $$\left(K_{\sigma }+K_{s}-K_{g}^{me}-\Delta TK_{g}^{th}\right)\;\bar{d}=0$$

The above equation then results in having a thermal buckling problem when computing the critical buckling temperature $\Delta T_{cr}$:

(36) $$\left(K-\Delta TK_{g}^{th}\right)\;\bar{d}=0$$

with

(37) $$K=K_{\sigma }+K_{s}-K_{g}^{me}$$

Note that $K_{g}^{me}$ is computed using Equation (29) and plays a role in reducing the structural stiffness matrix of the plate. Second, the critical buckling load (CBL) $N_{cr}$ for a given thermal load can be solved using a similar procedure:

(38) $$\left(K_{\sigma }+K_{s}-K_{g}^{th}-\lambda K_{g}^{me}\right)\;\bar{d}=0$$

(39) $$\left(K-\lambda K_{g}^{me}\right)\;\bar{d}=0$$

where $K=K_{\sigma }+K_{s}-K_{g}^{th}$. Here, $K_{g}^{th}$ is also computed using Equation (29) with $N_{\alpha \beta }$, which is computed using thermally induced stress.

In the above Equation (28), ${\hat{D}}_{s}$ indicates the modified shear modulus matrix given by

(40) $${\hat{D}}_{s}=\frac{\kappa \;G}{1+\vartheta \cdot {\left(L_{e}/h\right)}^{2}}\left[\begin{array}{cc} 1 & 0\\ 0 & 1 \end{array}\right]$$

where $L_{e}$ is the longest side length of a Delaunay triangle. This modification is introduced to further stabilize shear locking suppression through the MITC approach by adjusting a shear stabilization parameter $\vartheta \left(\vartheta >0\right)$ [38].

## 5. Results and Discussion

The numerical integrations of the stiffness matrices and the thermal load vector $F_{th}$ in Equations (27)–(30) are performed via seven-point Gaussian integration over the triangular mid-surface, with the trapezoidal rule applied to 50 uniform segments across the thickness. Meanwhile, the mechanical load vector $F_{me}$ in Equation (31) is numerically integrated by applying two Gauss points to the plate edges. Two kinds of boundary conditions (BCs), clamped (c) and simply supported (S), are enforced, and the clamped condition is enforced as

(41) $$C:\;u_{0}=v_{0}=\omega_{0}=\vartheta_{x}=\vartheta_{y}=0$$

The simply supported condition is

(42) $$S:\;v_{0}=\omega_{0}=\vartheta_{y}=0$$

at x=0 and a in the FSDT. The displacement components $v_{0}$ and $\vartheta_{y}$ in Equation (40) are changed to $u_{0}$ and $\vartheta_{x}$ for the sides at y=0 and b. The shear stabilization parameter ϑ in Equation (40) is set to 0.1, as recommended by Lyly et al. [39].

**Figure 4:**
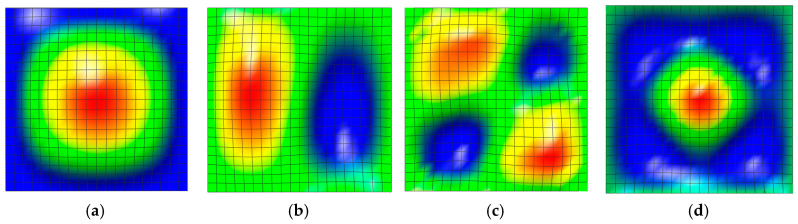
Lowest-mode shapes in the bi-axial mechanical buckling: (**a**) first, (**b**) second, (**c**) third, and (**d**) fourth.

First, clamped isotropic epoxy plates were used to investigate whether the test program provided a converged value as the grid density increased. The geometry dimensions of the plate were a=b=0.1 m and h=0.02 m, and the material properties were E=70 GPa, ν=0.3 and $\alpha =23\times 10^{-6}/K$, respectively. Uni-axial mechanical buckling and thermal buckling due to a uniform temperature rise were separately performed, and the numerical results are given in Table 1. The CBL $N_{cr}$ and the CBTR $\Delta T_{cr}$ were calibrated as ${\bar{N}}_{cr}=aN_{cr}/\left(Eh^{2}\right)$ and $\Delta {\bar{T}}_{cr}=\alpha \Delta T_{cr}/\left(h/a\right)$, respectively. The relative differences ${\bar{N}}_{cr}^{rel}\left(\%\right)$ and $\Delta {\bar{T}}_{cr}^{rel}\left(\%\right)$ were calculated as $\left|A-\hat{A}\right|/\hat{A}\times 100\%$, with $\hat{A}$ being the numerical value obtained using a grid density of 23×23. A grid density of 21×21 shows relative differences of less than 1.0%, so this grid density was chosen for all numerical experiments in this study. Figure 4 presents the shapes of the four lowest bi-axial buckling modes of the clamped isotropic epoxy plate, which were obtained using the 21×21 uniform NEM grid. The differences among the four buckling modes can be clearly observed.

Next, uncoupled thermal and mechanical buckling analyses were performed for the GPL-reinforced epoxy plate by changing the GPL mass fractions $g_{GPL}^{*}$ and the GPL dispersion types. The material properties of the underlying epoxy matrix were the same as those of the previous example, and the sizes and the material properties of the GPL were as follows [40]: $w_{GPL}=1.5\;\mu m,$ $t_{GPL}=1.5\;\;nm,$ $E_{GPL}=1.01\;TPa,\;\nu_{GPL}=0.186$, and $\alpha_{GPL}=5.0\times 10^{-6}/K$, respectively. Note that the material properties of the GPL and matrix were assumed to be the values at room temperature, and their dependences on temperature and displacement were not considered. The above clamped plate with the same geometric dimensions was chosen, and the computed non-dimensional CBTRs $\Delta {\bar{T}}_{cr}$ and buckling loads ${\bar{N}}_{cr}$ are presented in Table 2. With mechanical buckling, the CBL increased in proportion to the GPL mass fraction $g_{GPL}^{*}$, and the bi-axial buckling exhibited a buckling load nearly half that of the uni-axial buckling. The CBL was found to be affected by the GPL dispersion type, such that the highest levels appeared in the FG-X, the lowest in the FG-O, and the second and third levels were in the FG-U and FG-Λ, respectively. Thermal buckling also showed a similar trend with respect to the GPL dispersion type, such that the FG-X and FG-O exhibited the highest and lowest levels, respectively. This implies that the magnitude of structural stiffness is ordered as follows: FG-X, FG-U, FG-∧, and FG-O. However, the variation in the CBTR with respect to the GPL mass fraction depends on the GPL dispersion type. The FG-U and FG-X show uniform increases in the CBTR with the GPL mass fraction, but the FG-O and FG-∧ showed the reverse trend. This is because thermal buckling is affected not only by the structural stiffness but also by the thermal expansion coefficient, and the thermal expansion coefficient of the GPL was smaller than that of the epoxy, unlike the modulus of elasticity. Therefore, the thermal buckling stiffness of the FG-GPLRC epoxy plate depended on the relative variation with respect to the GPL dispersion type between the elastic modulus and the thermal expansion coefficient.

In the next step, the buckling of the FG-GPLRC epoxy plate under coupled thermal and mechanical loads was examined, with a focus on the key parameters. The geometric dimensions and boundary conditions of the plate matched those in the previous example, unless otherwise specified. The buckling analysis simulation procedure under the coupled thermal and mechanical loads was as follows. Thermal and mechanical buckling analyses were performed separately, and the mechanical buckling load $N_{cr}$ was divided into the desired number of uniform segments. Next, the CBTRs $\Delta T_{cr}$ corresponding to each discretized mechanical buckling load were obtained through thermal buckling analyses, in which the discretized mechanical buckling loads were considered.

**Figure 5:**
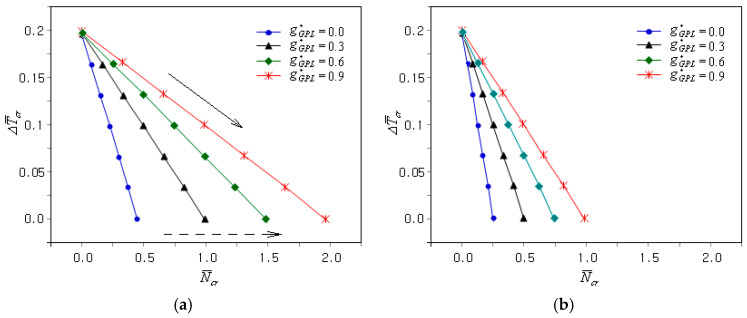
Influence of the GPL mass fraction $g_{GPL}^{*}$ on the coupled thermo-mechanical buckling of the clamped FG-U GPLRC plate: (**a**) γ=0; (**b**) γ=1.

Figure 5a comparatively represents the ${\bar{N}}_{cr}$ − $\Delta {\bar{T}}_{cr}$ plots of the clamped FG-GPLRC epoxy plate under uni-axial mechanical buckling (i.e., γ=0). The CBTR uniformly decreased proportionally to the mechanical buckling load, as indicated by a solid arrowed line. This is because the buckling stiffness of the plate weakened as the mechanical buckling load was applied. The variation in the CBTR without the mechanical buckling load with respect to the GPL mass fraction was negligible, as shown in Table 2. However, the slope of the ${\bar{N}}_{cr}$ − $\Delta {\bar{T}}_{cr}$ plots became gentler as the GPL mass fraction increased, as indicated by the dotted arrowed line, because the CBL increased with the increase in the GPL mass fraction for the FG-U distribution pattern. Figure 5b shows the ${\bar{N}}_{cr}$ − $\Delta {\bar{T}}_{cr}$ plots for the bi-axial mechanical buckling, where the plot slope changes.

**Figure 6:**
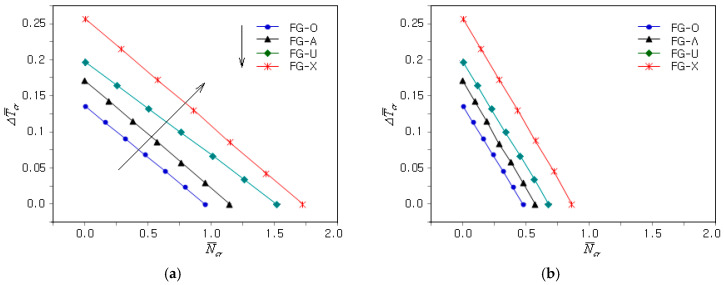
Influence of the GPL dispersion type on the coupled thermo-mechanical buckling of the clamped FG-GPLRC plate: (**a**) γ=0, (**b**) γ=1.

Figure 6a shows the effect of the GPL dispersion type on the coupled thermo-mechanical uni-axial buckling of the FG-U clamped GPLRC plate. First, it can be seen that the ${\bar{N}}_{cr}$ − $\Delta {\bar{T}}_{cr}$ plots are quite different from those in Figure 5a. As shown in Table 2, the CBTR corresponding to the zero CBL was markedly affected by the GPL dispersion type. The slope of the ${\bar{N}}_{cr}$ − $\Delta {\bar{T}}_{cr}$ plot varies depending on the GPL dispersion type, such that the slope of the FG-U is the gentlest, while the other three are very similar. The magnitude of the ${\bar{N}}_{cr}$ − $\Delta {\bar{T}}_{cr}$ plot was the highest for the FG-X and the lowest for the FG-O, with the FG-U and FG-Λ ranking second and third, respectively, as indicated by the solid arrowed lines. This indicates that the coupled thermo-mechanical buckling stiffness of the FG-GPLRC plates follows this order. Figure 6b represents the ${\bar{N}}_{cr}$ − $\Delta {\bar{T}}_{cr}$ plots when the uni-axial mechanical buckling was changed to bi-axial. One can see that the buckling load level decreased uniformly without any change in the CBTR.

**Figure 7:**
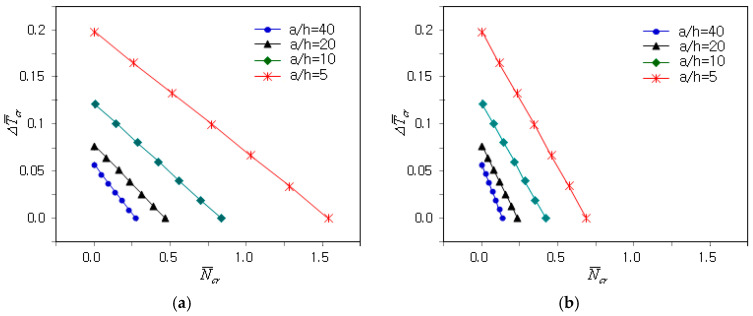
Influence of the plate’s relative thickness ratio on the coupled thermo-mechanical buckling of the clamped GPLRC plate: (**a**) γ=0; (**b**) γ=1.

Figure 7a shows the influence of the plate’s relative thickness ratio a/h on the ${\bar{N}}_{cr}$ − $\Delta {\bar{T}}_{cr}$ plots of the clamped FG-U and clamped GPLRC plates when γ is zero (i.e., uni-axial mechanical buckling). It can be observed that both the CBTR corresponding to the zero mechanical buckling load and the slope of the ${\bar{N}}_{cr}$ − $\Delta {\bar{T}}_{cr}$ plot were significantly affected by the plate relative thickness ratio. This was due to the significant decrease in the coupled thermo-mechanical buckling stiffness of the FG-GPLRC plate as the plate’s relative thickness ratio increased. The ${\bar{N}}_{cr}$ − $\Delta {\bar{T}}_{cr}$ plots are different because the changes in the thermal and mechanical buckling stiffness relative to the plate thickness ratio are not equal. The ${\bar{N}}_{cr}$ − $\Delta {\bar{T}}_{cr}$ plots for bi-axial mechanical buckling are comparatively represented in Figure 7b, showing that the buckling load level decreased uniformly as the CBTR remained unchanged.

**Figure 8:**
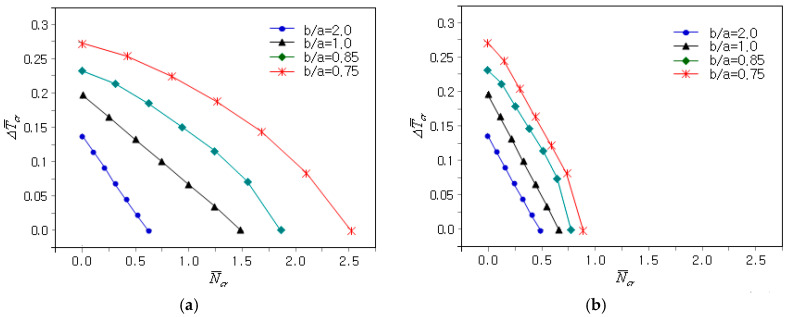
Influence of the aspect ratio of the plate on the coupled thermo-mechanical buckling of the clamped FG-U GPLRC plate: (**a**) γ=0.0; (**b**) γ=1.0.

Figure 8a presents the effect of the plate’s aspect ratio b/a on the coupled thermo-mechanical buckling of the clamped FG-U GPLRC plate when γ is 0. First, the relationship between ${\bar{N}}_{cr}$ and $\Delta {\bar{T}}_{cr}$ became nonlinear as the value of b/a became less than unity. This implies that the interdependence of the thermal expansion coefficient and the elastic modulus varied depending on the plate’s aspect ratio. Second, both ${\bar{N}}_{cr}$ and $\Delta {\bar{T}}_{cr}$ decreased with an increasing b/a because the coupled thermo-mechanical buckling stiffness decreased proportionally to the b/a value. Figure 8b represents a comparison of the ${\bar{N}}_{cr}$ − $\Delta {\bar{T}}_{cr}$ plots for bi-axial mechanical buckling, showing that the CBL decreased uniformly as the CBTR remained unchanged.

**Figure 9:**
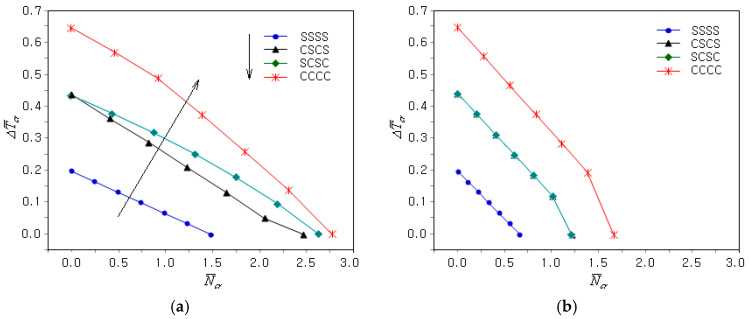
Influence of the boundary conditions on the coupled thermo-mechanical buckling of the FG-U GPLRC plate: (**a**) γ=0; (**b**) γ=1.

Figure 9a shows the influence of the boundary condition on the ${\bar{N}}_{cr}$ − $\Delta {\bar{T}}_{cr}$ plots of the uni-axial coupled thermo-mechanical buckling of the FG-U GPLRC plate. In the figure, four-letter acronyms consisting of C and S indicate the boundary conditions applied to the four sides ①, ②, ③ and ④ of the plate, as depicted in Figure 2. First, the effect of the boundary condition appears to be higher on $\Delta {\bar{T}}_{cr}$ than on ${\bar{N}}_{cr}$, and it is the highest at CCCC and the lowest at SSSS, with SCSC and CSCS ranking second and third being, as indicated by the solid arrowed line. This is because the clamped boundary condition was more intensive than the simply supported condition and the buckling stiffness increased proportionally to the boundary condition intensity. Figure 9b shows the change in the ${\bar{N}}_{cr}$ − $\Delta {\bar{T}}_{cr}$ plots when uni-axial mechanical buckling was replaced by bi-axial mechanical buckling. The difference between CSCS and SCSC disappeared, and the CBTR remained unchanged, leading to a uniform reduction in the mechanical buckling loads. This is because uni-axial and bi-axial buckling only affects the magnitude of the mechanical buckling load.

Lastly, the coupled thermo-mechanical buckling of the GPLRC plate was compared with that of the CNTRC plate by using the same underlying matrix. The (10,10) single-walled CNT was chosen [41], and its orthotropic elastic properties are given in Table 3, together with the thermal expansion coefficients $\alpha_{11}=3.4584\times 10^{-6}/K$ and $\alpha_{22}=5.1682\times 10^{-6}/K$. According to the modified rule of mixtures, the effective elastic moduli of the CNTRC plate were determined by

(43) $$E_{1}=\eta_{1}V_{cnt}E_{1}^{cnt}+V_{m}E_{m},\;\;\frac{\eta_{2}}{E_{2}}=\frac{V_{cnt}}{E_{2}^{cnt}}+\frac{V_{m}}{E_{m}}$$

(44) $$\frac{\eta_{3}}{G_{12}}=\frac{V_{cnt}}{G_{12}^{cnt}}+\frac{V_{m}}{G_{m}}$$

where $\eta_{i}\left(i=1,2,3\right)$ are the CNT efficiency parameters depending on the CNT volume fraction $V_{cnt}^{*}$ [42]. The effective orthotropic thermal expansion coefficients and Poisson’s ratios were calculated by the simplest linear rule of mixtures [5].

**Figure 10:**
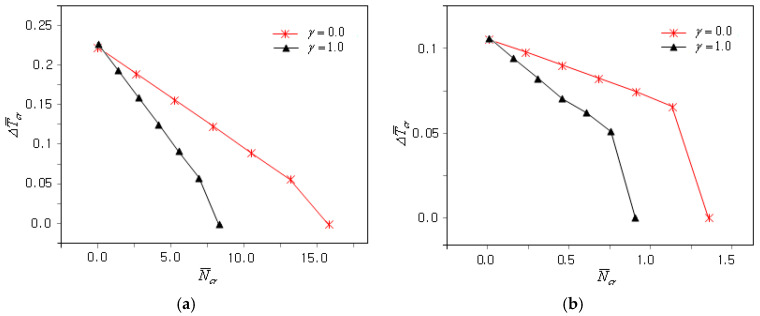
Uni- and bi-axial coupled thermo-mechanical buckling (FG-U, $V_{GPL}^{*}=V_{CNT}^{*}=0.12$): (**a**) FG-GPLRC; (**b**) FG-CNTRC.

Figure 10a,b comparatively present the ${\bar{N}}_{cr}$ − $\Delta {\bar{T}}_{cr}$ plots for the clamped FG-U GPLRC and CNTRC plates, respectively, where both the GPLRC and CNTRC plates show clear differences in the plots. In addition, it can be observed that the GPLRC plates had a significantly higher CBTR $\Delta {\bar{T}}_{cr}$ and buckling load ${\bar{N}}_{cr}$ compared to the CNTRC plates for the same volume fraction $V_{GPL}^{*}=V_{CNT}^{*}=0.12$. These detailed numerical data indicate that the relative ratios between the GPLRC and CNTRC plates were 2.11 in the CBTR and 9.02 and 11.56 in the uni- and bi-axial mechanical buckling loads, respectively. These comparison results clearly demonstrate that the thermo-mechanical buckling stiffness of the GPLRC plate was much higher than that of the CNTRC plate. This is because the introduction of the CNT efficiency parameters $\eta_{i}$, which were much smaller than unity in Equations (43) and (44), led to the effective elastic modulus of the CNTRC being smaller than that of the GPLRC for the same volume fraction.

## 6. Conclusions

This study investigated the buckling of a FG-GPLRC plate under coupled thermal and mechanical loads using a newly developed numerical method. The coupled buckling problem was formulated according to the minimum potential energy theorem, and the buckled deformation was expressed by the FSDT. The formulated problem was approximated by the 2-D natural element method to derive the eigen matrix equations for computing the CBTR and the CBL. Convergence and benchmark tests were carried out to examine the reliability of the developed numerical method. Additionally, the coupled thermo-mechanical buckling behavior was parametrically investigated with respect to the key parameters, together with a comparison with the FG-CNTRC plate. The results lead to the following key observations:

- The developed 2-D NEM-based numerical method demonstrated stable convergence with a relative difference equal to 1.0% at a fine grid density.
- The CBTR decreased in proportion to the in-plane compressive load, while the CBL decreased proportionally to the uniform temperature rise.
- The replacement of uni-axial compression with bi-axial compression almost halved the CBL, but without affecting the CBTR.
- The increase in the GPL mass uniformly increased the CBL without changing the CBTR and the linear relationship between ${\bar{N}}_{cr}$ and $\Delta {\bar{T}}_{cr}$.
- The GPL dispersion type and the boundary condition affected both the CBTR and the CBL, while keeping the linear relationship between ${\bar{N}}_{cr}$ and $\Delta {\bar{T}}_{cr}$. The magnitude orders in the critical values were as follows: FG-X > FG-U > FG-Λ and FG-O, and CCCC > SCSC > CSCS > SSSS, respectively.
- The increase in the relative thickness ratio and the aspect ratio increased both the CBTR and the CBL. The former did not affect the linear relationship between ${\bar{N}}_{cr}$ and $\Delta {\bar{T}}_{cr}$, but the latter altered the linear relationship when it was small.
- Compared to the GPLRC plate, the CNTRC plate showed different ${\bar{N}}_{cr}$ − $\Delta {\bar{T}}_{cr}$ plots and much smaller CBTR and uni- and b-axial buckling loads.

The present study was limited to simple intact structures. But almost all real structures are complex and damaged, so the current study needs to be extended to these structures, such as cylindrical panels with internal cracks. In addition, the equivalent shear modulus estimated by the isotropic Halpin–Tsai model is known to be underestimated when the thickness is not small [43], so it is necessary to investigate the changes in the results using a material property estimation model. This presents a valuable research topic for future work.

## Figures and Tables

**Table 1:** Convergence of the test program to the grid density for a clamped isotropic epoxy plate (a/b=1, a/h=20,ν=0.34).

Items	Grid Density (N × N)						
	11 × 11	13 × 13	15 × 15	17 × 17	19 × 19	21 × 21	23 × 23
${\bar{N}}_{cr}$	0.2477	02353	0.2283	0.2240	0.2211	0.2193	0.2179
${\bar{N}}_{cr}^{rel}\left(\%\right)$	13.676	7.985	4.773	2.799	1.459	0.642	-
$\Delta {\bar{T}}_{cr}$	0.2167	0.2083	0.2033	0.2000	0.1978	0.1959	0.1937
$\Delta {\bar{T}}_{cr}^{rel}\left(\%\right)$	10.374	6.585	4.330	2.842	1.849	0.992	-

**Table 2:** The CBTRs $\Delta {\bar{T}}_{cr}$ and the CBLs ${\bar{N}}_{cr}$ of the clamped FG-GPLRC epoxy plate (a/b=1, a/h=20,ν=0.34).

Type of Buckling		$g_{GPL}^{*}\left(\%\right)$	GPL Dispersion Type			
			FG-U	FG-O	FG-X	FG-∧
Thermal		0.0	0.1950	0.1950	0.1950	0.1950
		0.4	0.1959	0.1437	0.2469	0.1763
		0.8	0.1969	0.1298	0.2621	0.1649
Mechanical	γ=0	0.0	0.4387	0.4387	0.4387	0.4387
		0.4	1.0209	0.7633	1.2593	0.9252
		0.8	1.6027	1.0820	2.0740	1.3568
	γ=1	0.0	0.2193	0.2193	0.2193	0.2193
		0.4	0.5105	0.3816	0.6295	0.4625
		0.8	0.8013	0.5410	1.0369	0.6784

**Table 3:** Material properties of (10,10) single-walled CNTs (1,2,3=x,y,z).

Elastic Moduli (GPa)			Poisson’s Ratios			Shear Moduli (GPa)		
$E_{1}^{cnt}$	$E_{2}^{cnt}$	$E_{3}^{cnt}$	$\nu_{12}^{cntT}$	$\nu_{23}^{cnt}$	$\nu_{31}^{cnt}$	$G_{12}^{cnt}$	$G_{23}^{cnt}$	$G_{31}^{cnt}$
5646.6	7080.0	-	0.175	-	-	1944.5	-	-

## Data Availability

The original contributions presented in the study are included in the article. Further inquiries can be directed to the corresponding author.
